# A Study of the Interactions of Heavy Metals in Dairy Matrices Using Fourier Transform Infrared Spectroscopy, Chemometric, and In Silico Analysis

**DOI:** 10.3390/foods12091919

**Published:** 2023-05-08

**Authors:** Alfredo C. Benítez-Rojas, María E. Jaramillo-Flores, Orlando Zaca-Moran, Israel Quiroga-Montes, Raúl J. Delgado-Macuil

**Affiliations:** 1 Centro de Investigación en Biotecnología Aplicada, Instituto Politécnico Nacional, Santa Inés Tecuexcomac 90700, México; alfcesar@gmail.com (A.C.B.-R.); ozacam@ipn.mx (O.Z.-M.); 2 Ingeniería Bioquímica, Escuela Nacional de Ciencias Biológicas (ENCB), Instituto Politécnico Nacional, México City 07738, México; mjarfl@ipn.mx; 3 Universidad Popular Autónoma del Estado de Puebla (UPAEP) A.C., 21 sur #1103, Barrio de Santiago, Puebla 72410, México; israel.quiroga@upaep.mx

**Keywords:** FTIR, heavy metal, casein, milk, dairy products

## Abstract

Heavy metals are among the toxic substances longest recognized by man. Today, due to the myriad sources of exposure, such as contaminated water, food, or air, they have become a major public health problem. This work presents the effects manifested in the infrared spectrum behavior caused by the presence of Cd^2+^, Cr^6+^, and Pb^2+^ at different concentrations in three different matrices: water, casein, and milk; observing that the spectral modifications in the regions of different vibrational modes of nucleophilic groups such as -OH, COO- and NH_2_ depending on the nature of the metal and its concentration. These findings were correlated in-silico using optimized models in Gabedit software and structural optimization was performed with MOPAC 2016 showing stable structures between the metals and Gln, Hys, Glu, and Phe of casein. By applying chemometrics (Principal Component Analysis), it was possible to observe a good correlation between the experimental data and to discriminate between the type of metal, the matrix that contains it, and the concentration could be represented through linear models that showed adjustments with a value of r^2^ ≥ 0.95.

## 1. Introduction

Heavy metals (HM) are among the oldest toxic substances longest recognized by man, even at very low concentrations it causes serious damage to human health [1]. Currently, due to environmental contamination, their presence in water, food, or air makes it more likely that humans will be exposed to them, which is a major public health problem. The sources of generation of these elements are very diverse; the most important are soil and aquifer contamination, soil erosion, industrial discharges, and pesticides, among others [2]. This contamination chain usually follows a cycle: industry, atmosphere, soil, water, food, and man [3]. In developing countries, the problem is especially serious since high levels of HM especially Pb and Cd, have been reported in dairy products [4,5,6]. HM in dairy products may be due to contamination of the original cow’s milk, which may be due to exposure of lactating cows to environmental pollution or consumption of contaminated feeding stuff or water [5]. Milk constants consume or their subproducts (for example, cheese and yogurt) represent an important focus of HM bioaccumulation in human tissues [1,7,8].

For the above, food science and industry require inexpensive, accurate, reproducible, and preferably non-destructive rapid analytical techniques. Infrared (IR) Spectroscopy is a well-established technique for the identification and structural analysis of chemical compounds; the peaks in the IR spectrum represent the excitation of the vibrational modes of the molecules in the sample and are therefore associated with the various chemical bonds and functional groups present in molecules [9]. The IR spectrum of a compound is, therefore, one of its most characteristic physical properties and can thus be considered its “fingerprint” [10]. IR spectroscopy is also a powerful tool for quantitative analysis, as the amount of IR energy absorbed by a compound is proportional to its concentration. However, until recently, it has had rather limited application in both qualitative and quantitative analysis of food systems, largely due to experimental limitations [11]. Currently, many applications are based on instrumental analytical chemistry in the laboratory, coupled with chemometrics, for example, the use of partial least squares to calibrate the ultraviolet (UV)/visible spectrum of a mixture of pharmaceuticals helps determine the concentrations of each component. There are numerous types of similar applications in a wide variety of spectroscopies, such as IR (of all types), atomic spectroscopy, mass spectrometry, and in chromatography, such as High-Performance Liquid Chromatography (HPLC) and gas chromatography, and also mass spectrometry (GC-MS) [12]. This method, although perhaps not very glamorous, is still very widely used and most reports referring to the use of chemometrics in the academic literature refer to instrumental analytical chemistry, which is cited as very successful and often essential, especially concerning experimental design and interpretation of multivariate spectroscopic data [13]. Karl Pearson first formulated Principal Components Analysis (PCA) in the field of statistics in 1901, he formulated the analysis to “find lines and planes that most accurately fit to point systems in space.” In the 1930s, Thurstone and other psychologists pioneered the development of factor analysis (FA), mentioned here because FA is closely related to PCA and the two methods are often incorrectly confused. Since then, the utility of PCA has been rediscovered in various scientific fields, leading, among other things, to an abundance of redundant terminology. Eigenvalue analysis and eigenvector analysis are frequently applied to the physical sciences. In chemistry, in about 1960, Michael F. Malinowski introduced Principal Components (PC) known then as principal factor analysis, and since 1970 many chemical applications have been devised [14].

PCA principally aims to reduce the dimensionality of a data set, consisting of a large number of interrelated variables, while as far as possible maintaining the representativeness of the data set. This is achieved by transforming a new set of variables (principal components), which may or may not be correlated with each other, but are always ordered so that the first components retain most of the variation among all the original variables [11]. Currently, many works have been reported using Fourier-Transform Infrared Spectroscopy (FTIR spectroscopy) together with chemometric analysis to identify adulterations in food [15,16,17], and to distinguish some characteristics of interest in various matrices [10,18,19,20,21,22] and even quantify analytes of interest in complex matrices [23,24,25,26,27]. The application of IR spectroscopy for the quantitative determination of fat, protein, and lactose in milk has been an official method used by the Association of Official Agricultural Chemists (AOAC) since 1984 [28]. Commercial IR milk analyzers are widely used as the basis for milk quality control, dairy cattle registration, and routine quality control [29]. In most of these cases, organic compounds have been treated as targets for these determinations, however, some publications have already reported that it is also possible to observe and quantify the presence of inorganic compounds, in both organic and inorganic matrices, using FTIR spectroscopy [20,21,30,31,32,33,34,35,36,37,38,39]. Particularly in milk, chemometric analysis based on PCA, reveals determinate commercial parameters; such as quality and availability which together with consumers’ perception of milk [40], reveal differences between raw and commercial milk [41]. These are manifested in elemental differences, prior to specific detection of a variety of contaminants, antimicrobial residues [42], and bacterial or mold contamination [43,44]. Experimental data were obtained by infrared spectroscopy [39,42], UV/Vis spectroscopy [44], sensory tests [40], atomic absorption spectroscopy [41], or microbalance-based electronic noses [43]. Moreover, Gabedit is a freeware graphical user interface that offers preprocessing and postprocessing adapted to computational chemistry software packages. It includes tools for editing, displaying, analyzing, converting, and animating molecular systems [45]. In this work, it was used to study the interactions between heavy metal ions and the most abundant amino acids present in β-casein. This work aimed to determine the influence of heavy metals (Cd^2+^, Cr^6+^, and Pb^2+^) at different concentrations by examining the interactions between them in three matrices (water, casein, and milk) by IR spectroscopy; to finally correlate metal concentrations by PCA.

## 2. Materials and Methods

### 2.1. Reagents

Powdered CdCl_2_, Cr_2_O_3_, PbCl_2_, and β-casein were acquired from Merck. The dairy matrix (whole milk) in pasteurized form was obtained from a local brand (Santa Clara^®^ owned by the Coca-Cola^®^ group) and stored at 4 °C for preservation.

### 2.2. Heavy Metal Concentrations

Different solutions of Cd^2+^, Cr^6+^, and Pb^2+^ in water, β-casein, and milk were prepared using CdCl_2_, Cr_2_O_3_, and PbCl_2_ (Merck, Darmstadt, Germany). The concentration of HM in water ranged from 10^−4^ mgL^−1^ to 1000 mgL^−1^. β-Casein (Merck) was diluted in 10.5% aqueous solution, with metal concentrations ranging from 0.1 mgL^−1^ to 100 mgL^−1^. In the dairy matrix, concentrations of each metal ranged from 10^−3^ mgL^−1^ to 100 mgL^−1^. A very high concentration of casein was proposed because the study variable consisted of the concentration of HM. As the amount of protein and thus, the amount of amino acids present in the solution, were very high and constant in all systems, the conjecture was that this would better illustrate the interactions between the solutions and the HM, even at low metal concentrations.

### 2.3. Sample Preparation

**Figure 1:**
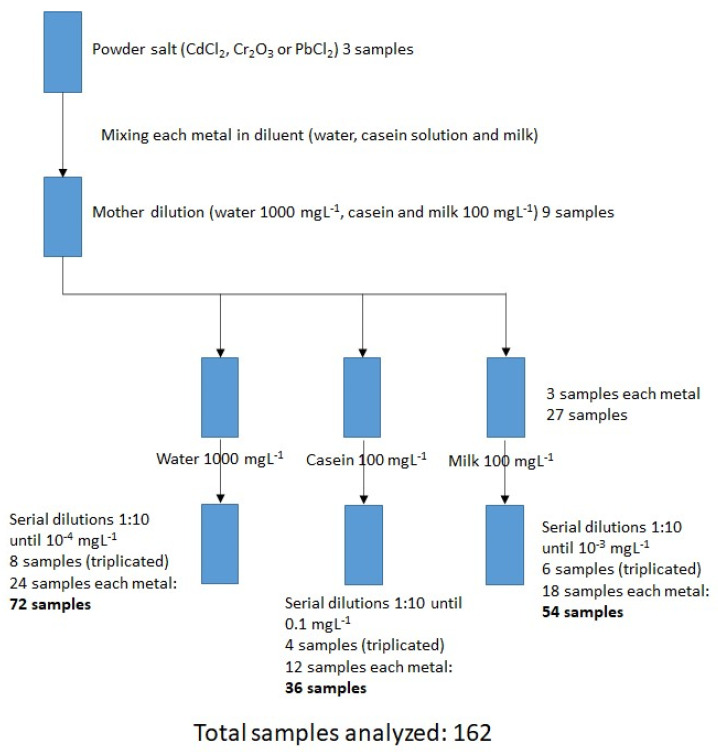
Schematic methodology for sample preparation in the three matrices.

Figure 1 presents the experimental design for sample preparation.

In water: Stoichiometric calculations were made from the solid salts, by preparing a stock solution of 1000 mgL^−1^ from each of the salts (CdCl_2_, Cr_2_O_3_, PbCl_2_) with deionized water as diluent. Subsequently, this was diluted by a factor of 10 until reaching the lowest concentration (10^−4^ mgL^−1^). In total, 24 dilutions were made with the 8 different concentrations (triplicate of each metal). Each sample was measured in triplicate in the FTIR equipment, depositing 5 µL of the sample in the reading glass, in order to obtain the raw spectra (absorbance vs wavenumber) and finally arrive at an average for the spectrum.

In casein: a 10.5% solution of β-casein in water was prepared to obtain the “diluent”. Stoichiometric calculations were made from the solid salts to prepare a 100 mgL^−1^ stock solution with each of the salts (CdCl_2_, Cr_2_O_3_, PbCl_2_) using the β-casein solution as diluent. Subsequently, this was diluted by a factor of 10, until reaching the lowest concentration (0.1 mgL^−1^). In total, 12 dilutions were made at the 4 different concentrations (triplicate of each metal). Each dilution was measured in triplicate in the FTIR equipment, depositing 5 µL of the sample in the reading glass, from which the raw spectra (absorbance vs wavenumber) were obtained, to finally obtain an average for the spectrum.

In milk: From the solid salts, stoichiometric calculations were made to prepare a stock solution of 100 mgL^−1^ with each of the salts (CdCl^2^, Cr_2_O_3_, PbCl_2_) using a commercial milk brand as diluent. Subsequently, this was diluted by a factor of 10, until the lowest concentration was reached (10^−3^ mgL^−1^). In total, 18 dilutions were made with the 6 different concentrations (triplicate for each metal). Each dilution was measured by triplicate in the FTIR equipment, depositing 5 µL of sample in the reading glass, from which the raw spectra (absorbance vs wavenumber) were obtained, to finally obtain an average for the spectrum.

Almost all the samples were analyzed immediately after their preparation, when necessary, they were stored for later analysis in a refrigerator at 4 °C. The storage and reading time never exceeded one day. Once the data was collected, the samples were discarded in a special container for HM, to be collected by a specialized company for further treatment.

### 2.4. Infrared Spectroscopy

Fourier Transform Infrared Spectroscopy in Attenuated Total Reflectance mode (FTIR-ATR) was acquired using a Vertex 70 spectrometer (Bruker, Billerica, MA, USA) at room temperature in the mid-infrared region, 4000 to 400 cm^−1^. 15 mL of each sample was collocated directly on the crystal in liquid form (previously prepared as in the sample preparation section mentioned) without any sample preparation and sampling immediately after the sample was put on the crystal (without any drying method), after that data was collected in attenuated total reflectance mode at a resolution of 4 cm^−1^. Each spectrum was obtained in triplicate and averaged, prior to analysis. The spectra were processed in Origin Pro 2016^®^ and the PCA was carried out in IBM SPSS version 22 software using the correlation method.

### 2.5. Data Analysis

Data analysis consisted of using the spectra numerically (absorbance vs. wavenumber) obtained experimentally. Firstly, by obtaining an average of the readings in triplicate, and secondly, by selecting specific regions, where the biggest differences in terms of absorbance intensity were associated with the heavy metal concentration; these spectra regions and combinations of them were entered into the Statistic Software SPSS for the purpose of PCA.

### 2.6. In-Silico Analysis

Three-dimensional molecular models were generated to elucidate the effects of interaction between casein and Cd^2+^, Cr^6+^, and Pb^2+^. The molecular models consisted of short chains and polypeptide chains that represent casein fragments with a high possibility of interaction between anion ions and one metal cation. The model structure was optimized using molecular dynamics conformational search in Gabedit software. The structure selected for each model was the one that presented the lowest energy. A second structural optimization and theoretical frequencies were performed using MOPAC 2016 software packages. The calculations for equilibrium structure search and theoretical frequencies were performed in water and with PM7 Hamiltonian.

## 3. Results

### 3.1. Spectra in Water

**Figure 2:**
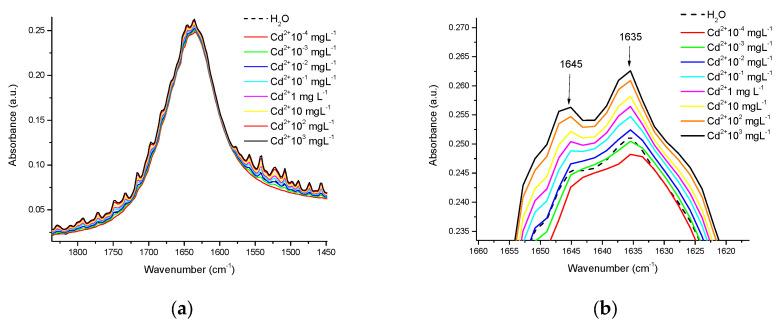
Disturbances in the scissoring region in the water spectra in the presence of Cd^2+^ at different concentrations of mgL^−1^: (**a**) Disturbances in the complete band of the scissoring movement, as a function of HM concentration; (**b**) Close-up of the highest peaks of this band.

In this work, we apply FTIR-ATR in the range of 4000–400 cm^−1^, coupled with chemometrics, to differentiate between the nature of the HM and the matrix in which it is placed (water, water-casein, or milk). In water, the measurements were made in crystal base mode, it was observed that the presence of Cd^2+^, Cr^6+^, and Pb^2+^ modifies the intensity of the “scissoring” band; Figure 2 presents an example of this, where in this region disturbances appear, and whose intensity is a function of the concentration in the presence of Cd^2+^ at several concentrations, ranging from 10^−4^ mgL^−1^ to 1000 mgL^−1^. Figure 2b presents a close-up of the highest peak in the scissor band, where the evident differences in absorbance are consistent with the metal concentration, initiating at a concentration of 10^−2^ mgL^−1^, with the highest peaks corresponding to the bands located at 1645 and 1365 cm^−1^, respectively.

**Figure 3:**
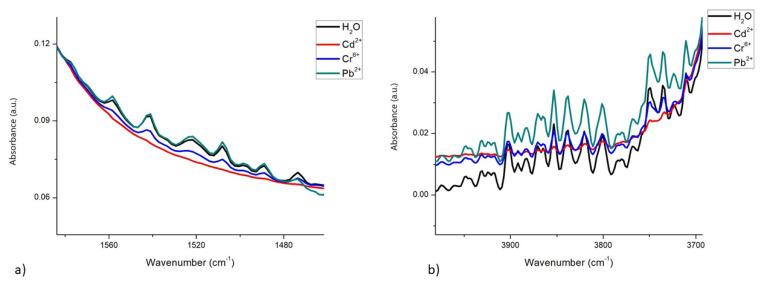
Differences in the spectra of water, with and without the presence of Cd^2+^, Cr^6+^, and Pb^2+^, at a concentration of 10^−4^ mgL^−1^: (**a**) In the scissoring region; (**b**) In the OH stretching region.

The spectral differences between metals are notorious even at concentrations as low as 10^−4^ mgL^−1^. Figure 3a shows a representation of the scissoring region, with the presence of the 3 metals at this concentration, revealing these differences. Figure 3b shows the differences in patterns between the spectra of the 3 metals in the OH stretching region (4000–3700 cm^−1^). These differences can be attributed to the interactions that each metal exerts on the scissoring and stretching movements of OH groups.

### 3.2. Spectra in β-Casein

**Figure 4:**
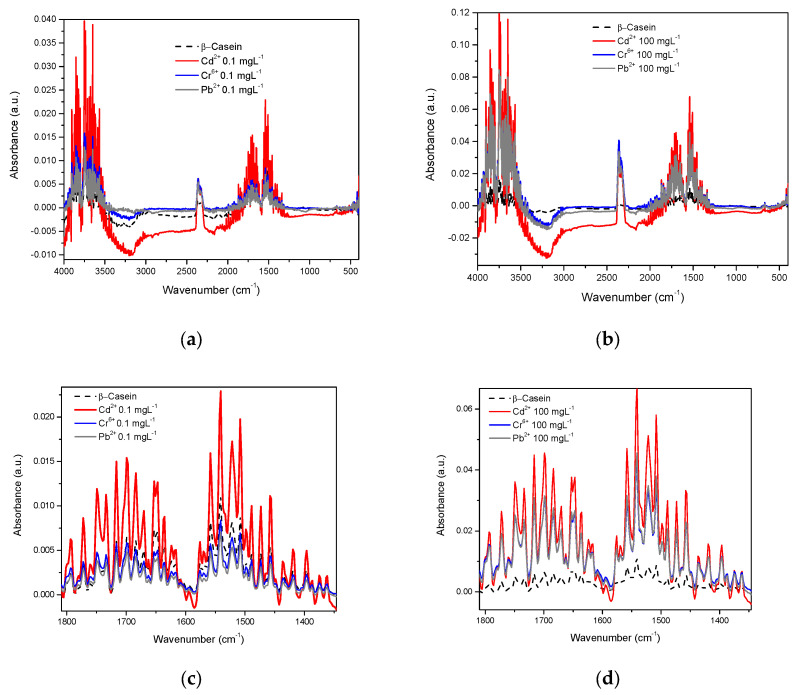
IR spectra of β-Casein in the presence of Cd^2+^, Cr^6+^, and Pb^2+^: (**a**) mid-infrared region at 0.1 mgL^−1^; (**b**) mid-infrared region at 100 mgL^−1^; (**c**) Amide region at 0.1 mgL^−1^; (**d**) Amide region at 100 mgL^−1^.

Considering both the β-casein and milk solutions, the spectra were acquired in the water-based mode (the water spectrum was eliminated). In the spectrum of β-casein, bands corresponding to the stretching vibration of the primary amide in its carboxyl groups between 1680 and 1660 cm^−1^ are observed, as well as the secondary amide between 1680 and 1640 cm^−1^. Bands corresponding to the bending vibration of the amino group are also visible in the primary amide between 1650 and 1620 cm^−1^ and between 1560 and 1530 cm^−1^; bands that represent NH bending vibrations and CN stretching of the secondary amide. Likewise, typical bands of both primary and secondary amides that correspond to different vibrational modes such as simple, symmetrical, and harmonic stretching of the amino and carboxyl groups also appear [46]. In this work, the spectra of β-casein with the presence of Cd^2+^, Cr^6+^, and Pb^2+^ were obtained at concentrations of 0.1 to 100 mgL^−1^ (Figure 4). In this image, it is apparent that the profiles of the complete spectra for each metal are very similar to each other (Figure 4a,b), however, evidently each profile is slightly different; at least enough to differentiate between them, if multivariate analysis techniques are applied (see chemometric analysis section). Figure 4c shows an approach to the region of amides, at low concentration (0.1 mgL^−1^) here absorbance intensities are very similar between pure casein, Cr, and Pb but the Cd spectrum presents visible differences, in comparison to 100 mgL^−1^, differences between each metal and casein are already clear (Figure 4d).

**Figure 5:**
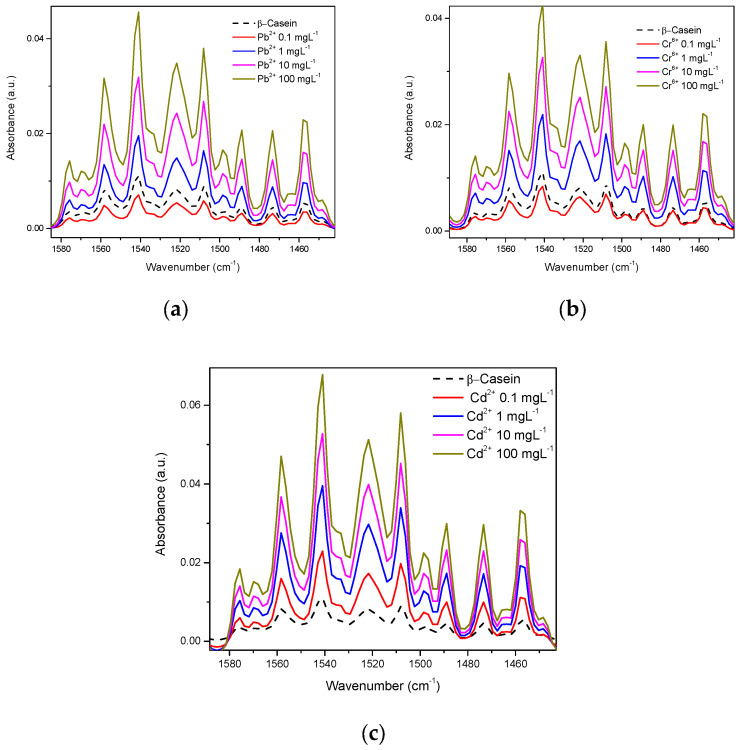
HM at different concentrations in the amide region in the casein spectrum: (**a**) Pb^2+^; (**b**) Cr^6+^; (**c**) Cd^2+^.

Figure 5 shows each metal mixed in β-casein solution at different concentrations in the amide region. A direct relationship exists between concentration and intensity of absorbance, except for at the lowest concentration (0.1 mgL^−1^) for the case of Pb^2+^ and Cr^6+^, however, for Cd^2+^, data are consistent for all concentrations tested (Figure 4c).

#### In-Silico Analysis

In-silico analysis revealed that absorbance peaks at experimentally measured wavenumbers correspond to bending vibrations of peptide bonds in specific geometries. Notably, the geometries described below (Table 1) may not be the only ones that absorb these wavenumbers. Furthermore, it was revealed that these geometries are not as stable if the casein fails to interact with an anion; as the charge repulsion between the multiple Glu residues curbs its stability. Therefore, these geometries can be achieved and stabilized, when HM such as Pb, Cd, and Cr interact with any of the multiple anionic groups; such as the Glu radical present in casein. The higher peak at 1540 cm^−1^ may be due to the ease with which the protein backbone forms these geometries at energy minima and due to these being the geometries stabilized when a Glu coordinates with a cation of any type.

**Figure 6:**
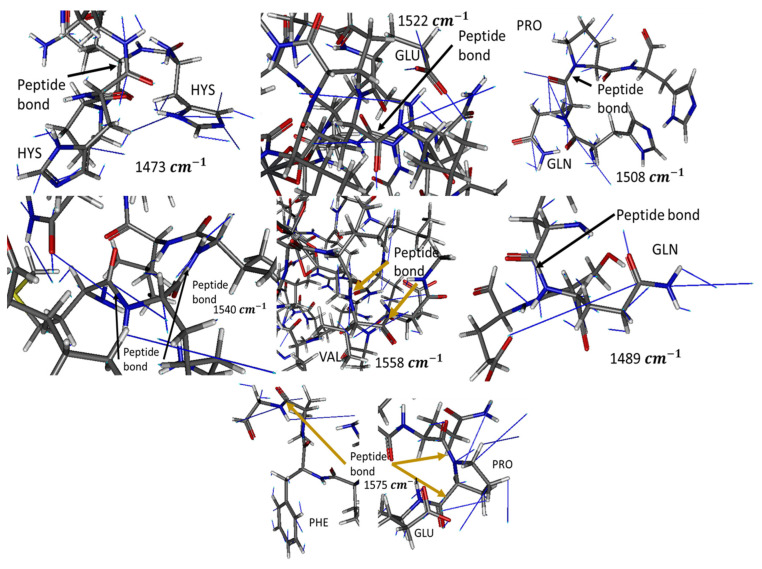
Examples of conformations of energetic minima, at which experimental absorbance can be observed. Blue lines show the vibrational paths of each atom at the corresponding wavelength. N is colored blue, C gray, O red, and H white.

Figure 6 shows the theoretical conformations of casein in which absorption peaks at different wavenumbers (resonances in the IR) were obtained. It describes the necessary geometries of the amine and amide groups for casein to obtain the experimental IR results. Thus, the presented in-silico model permits an understanding of how the concentration of HM reduces conformational changes in beta-casein and stabilizes its three-dimensional structure in the appropriate conformations to obtain the measured resonances. This reduction in conformational changes was because as the concentration of metals increases, the salt interactions between the metal cation and the anionic casein residues increase. This increases the number of theoretical conformations of casein in which experimental absorption peaks could be obtained in two ways: (1) it reduces the repulsion between the numerous anionic residues of casein and (2) stabilizes the protein backbone in the previously described conformations. In the milk industry, these interactions could reduce micellar stabilization and in consequence avoid milk subproducts generation.

### 3.3. Spectra in Milk

**Figure 7:**
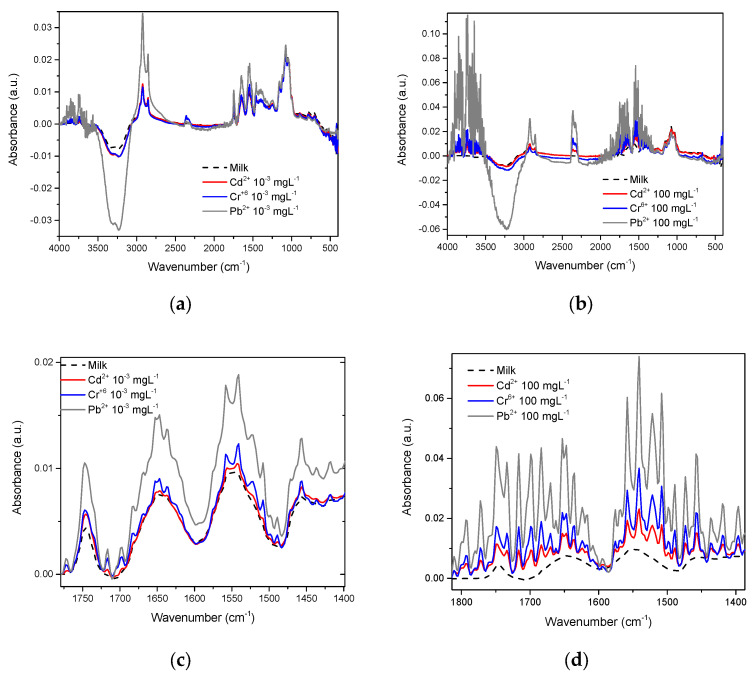
Whole milk spectra and approach to the amide region with the presence of Cd^2+^, Cr^6+^, and Pb^2+^: (**a**) Heavy metals at 10^−3^ mgL^−1^; (**b**) Heavy metals at 100 mgL^−1^; (**c**) Amide region at 10^−3^ mgL^−1^; (**d**) Amide region at 100 mgL^−1^.

The whole milk spectrum is reported by Iñon et al. [24]. In our work, the effects that Cd^2+^, Cr^6+^, and Pb^2+^ exert on this spectrum are presented, Figure 7 shows that in whole milk, even at concentrations as low as 10^−3^ mgL^−1^, it is also possible to observe differences between the spectra of each metal and milk (Figure 7a), such differences become more evident when the metal concentration increases to the maximum value tested in this study; 100 mgL^−1^ (Figure 7b). Just as observed with β-casein, in whole milk, the most intense vibrations were also found in the OH and amide regions. Conforming to this, we found notable differences between milk with these metals present and milk without metals (line dark) (Figure 7c,d).

**Figure 8:**
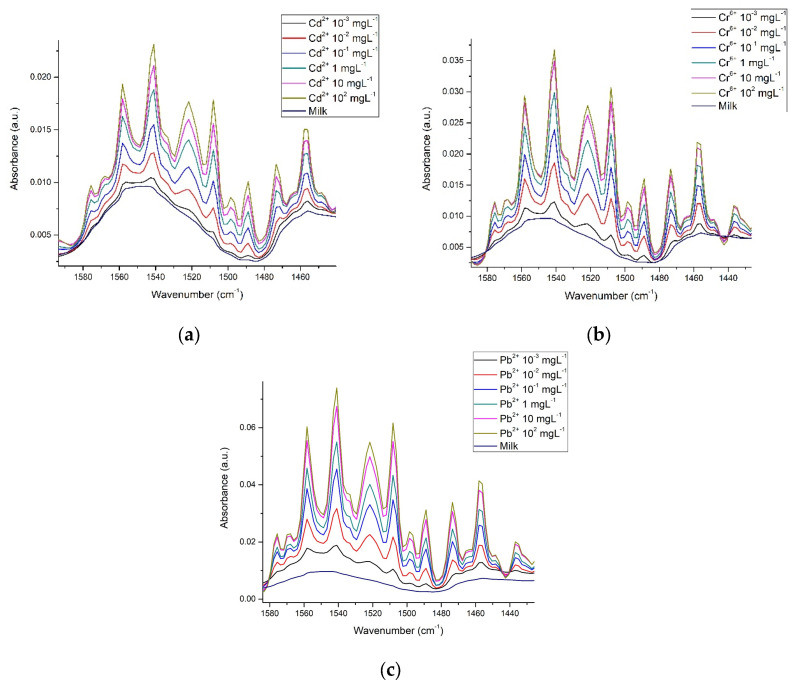
Spectra of milk in the amide region with the presence of (**a**) Cd^2+^; (**b**) Cr^6+^ and (**c**) Pb^2+^.

Figure 8 shows how in the amide region, absorbance intensities vary, depending on metal concentration. Here it is apparent that although the signals are very similar, there are slight differences between them; enough to make it possible, through multivariate analysis, to differentiate between each one of the metals, while conserving the relationship between metal concentration.

**Figure 9:**
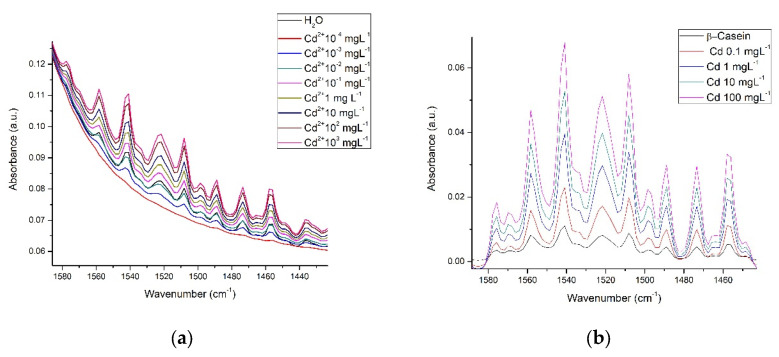

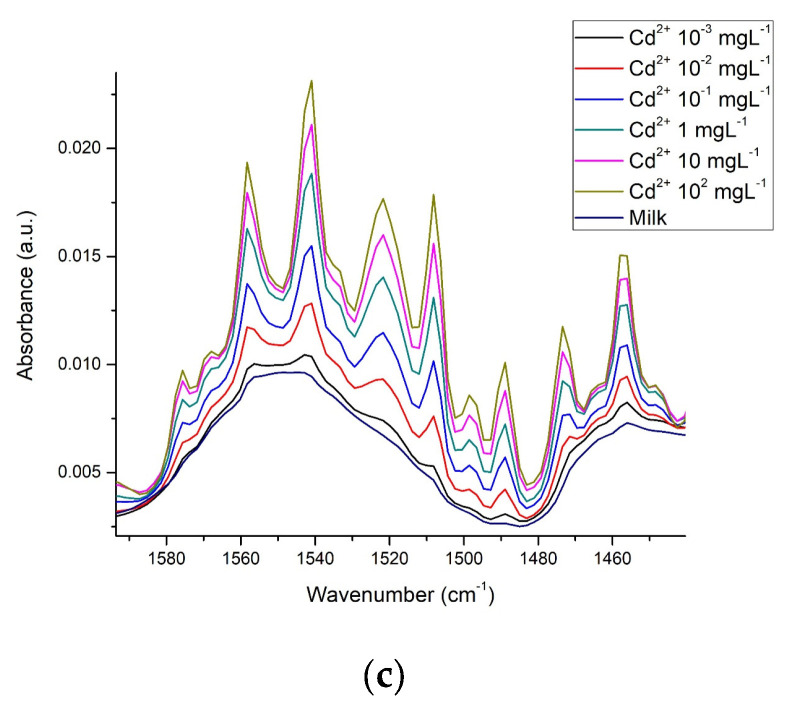
Cd^2+^ interactions in three different matrices at different concentrations: (**a**) Water in the scissoring region; (**b**) β-casein solution in the amide region; (**c**) Whole milk in the amide region.

Finally, we compare the three matrices; water, β-casein, and milk. Figure 9 shows the spectra that the presence of Cd^2+^ exerts on water (Figure 9a), β-casein solution (Figure 9b), and whole milk (Figure 9c), vibrations are consistent in the same region for the three matrices and absorbance intensity depends on cation concentration.

### 3.4. Chemometric Analysis

Data analysis consisted of using the spectra numerically (absorbance vs. wavenumber) obtained experimentally. Firstly, by obtaining an average of readings in triplicate, secondly, by selecting specific regions where the highest absorbance intensities were observed, depending on the concentration of the HM, see Table 2. These regions and their combinations were entered into SPSS to carry out the PCA, and finally, those that presented the best adjustments were selected and reported. The same treatment was carried out with casein and milk samples (Table 3). The spectra obtained were not normalized because it was considered that a minimum of data processing was required to preserve original variability and, thus, PCA results would be more aligned with the original data.

PCA mainly intends to reduce the dimensionality of a data set that consists of many interrelated variables [9]. This tool is widely used today for the processing of complex chromatograms and spectra with hundreds of values in the output signal (p.e. absorbance) while reducing dimensional space to a few points [10]. Multiple works have reported the use of chemometric tools, coupled to FTIR-ATR for discrimination and subsequent quantification of analytes of interest [10,16,17,23,24,25,26,51,52]. In this work, in all the tests, the first three main components (PC1, PC2, and PC3) explained 92% or more of the variability in the system; for PCA analysis the hydroxyl regions were used (3800–3000 cm^−1^, 1500–1450 cm^−1^), as well as the carboxyl and amide regions (1700–1400 cm^−1^).

**Figure 10:**
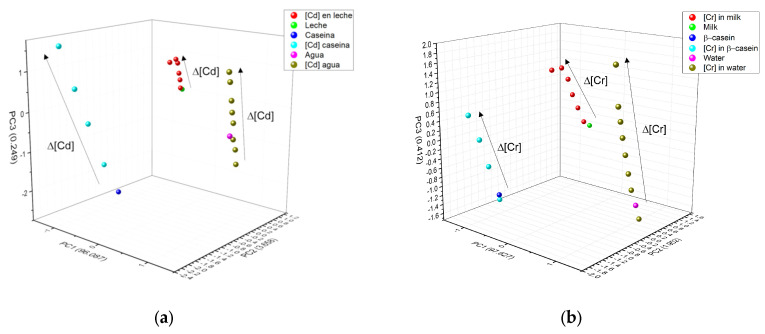

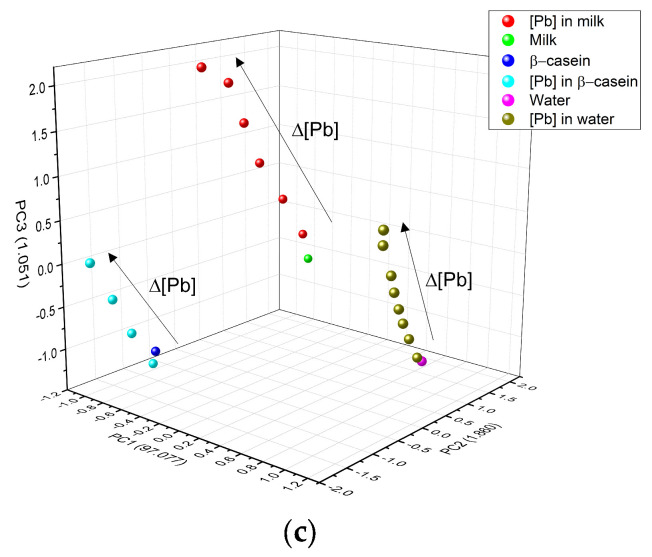
PCA and how it interacts in the three matrices: (**a**) Cd^2+^; (**b**) Cr^6+^ and (**c**) Pb^2+^.

Figure 10 shows the clustering of the 3 main components of the three metals (Figure 10a) Cd^2+^, (Figure 10b) Cr^6+^, and (Figure 10c) Pb^2+^. In all figures, water is colored pink, casein dark blue, and milk green. A completely linear trend can be observed, depending on metal concentration (arrow direction illustrates concentration increase), confirming that the type of matrix containing the metal can also be distinguished. The numerical data for metals that appear in β-casein is colored cyan, in milk red, and water olive green. In PCA, clustering according to the type of metal was well determined; it was also possible to identify any matrix that did not contain metal. The three HM present a linear relationship between concentrations tested, in the case of Cd^2+^, PCA analysis separated each concentration of the matrix individually (Figure 10a), whereas in the case of milk (red color), the concentrations did not separate so well and the highest ones remained very close; in the case of water for the same metal, there was a separation between concentrations, but the lowest ones were located very near to that of water without cadmium (pink color). Similar trends were observed with Cr^6+^ and Pb^2+^, where PCA was better able to separate these metals in milk (Figure 10b,c). As in the three metals, PC1 increases as a function of concentration, this component was associated with this experimental parameter, PC2 was associated with the type of matrix and PC3 was associated with the nature of the HM, as differences, which depend on the type of metal, can be observed at the surface.

**Figure 11:**
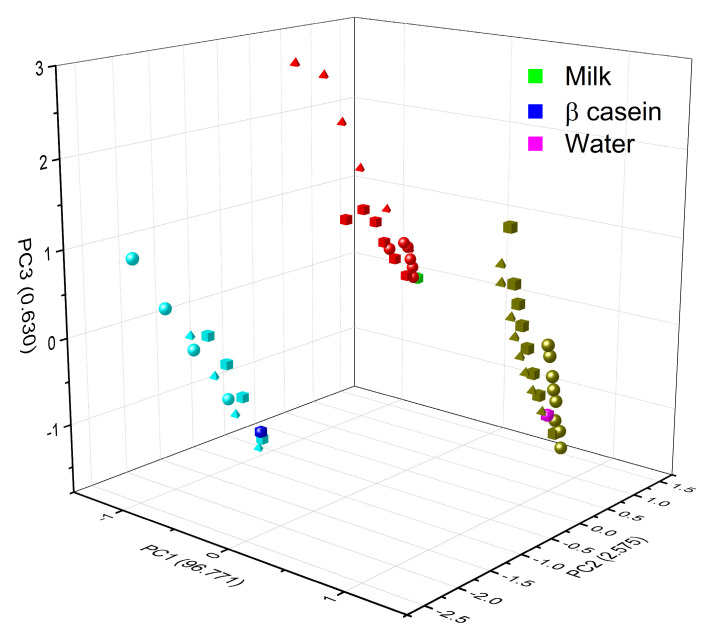
Grouping of PCA by type of matrix (blue casein, green milk, and magenta water) according to the concentration of the three different HM (Cd^2+^, Pb^2+^, and Cr^6+^). Circles are for Cd, squares are for Cr, and triangle are for Pb.

Figure 11 shows how the PCA was also able to very effectively separate the type of matrix studied; for this figure, all the experimental data for a specific matrix (β-casein in dark blue, milk in green and water in magenta) and the HM (in light blue are the HM in β-casein, in red those in milk, and olive green those in water) were added to the PCA analysis; maintaining the clustering according to the type of metal (circles are for Cd, squares are for Cr and triangle are for Pb) and a linear trend among concentrations, the lowest metal concentrations in each matrix are closer to the matrix without any metal present, and subsequently, they follow the linear tendency, depending on the type of metal and these are well distinguished.

**Figure 12:**
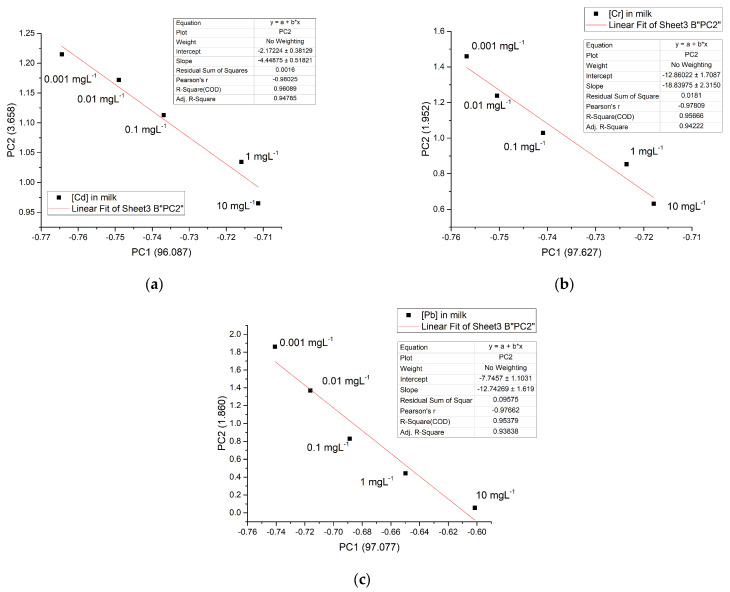
A linear relationship between PC1 and PC2 in milk matrix for the 3 metals studied: (**a**) Cd^2+^; (**b**) Cr^6+^, and (**c**) Pb^2+^.

The above information, PC1, and PC2 can be used to illustrate a relationship that makes it possible to correlate the concentration of metals in any of the matrices studied. Figure 12 shows the linear relationship between PC1 and PC2, depending on the concentration of metals in milk, with a range of 0.001 mgL^−1^ to 10 mgL^−1^, which is the concentration range of greatest interest for this type of metal and which are difficult to measure using other methods. Similar results were obtained for the casein and water solution (data not shown). The linear regression curve for both Cd^2+^ and Cr^6+^ manifested an adjustment of r^2^ = 0.96 and 0.95 for Pb^2+^, respectively.

## 4. Discussion

Many liquids have significant IR absorbance, but water is a bit more problematic, displaying broad, intense bands throughout the mid-infrared region. In this region, two intense bands are observed around 3500 cm^−1^ and 1636 cm^−1^, caused by the O-H stretching movement and the O-H-O bond scissors movement, respectively (see Figure 2 and Figure 3). The band around 4000–3000 cm^−1^ is very wide due to hydrogen bonds between water molecules and ions or osmolytes [32]. In addition to this, we located a smaller band centered at 1636 cm^−1^, which is the result of the coupling between scissor flexion movements and wide libration mode [47,53,54,55]; libration mode is a combination band associated with the presence of the H-bonding effect, in chemical and biological solute and cosolvent matrix [52]. The disturbances reported in this work in the spectrum of water with the presence of HM may be due to interactions exerted by metal ions when they are obstructing the natural “scissoring” movement of water molecules. Similar interactions with OH groups caused by the presence of cations of diverse nature, including HM, observed by FTIR have been reported [32,36,37]. Chen et al. [36] reported similar peaks in the corresponding wavenumber under the O-H elastic stretching bands in their spectra (at approximately 3580 cm^−1^), which were assigned to weakly hydrogen-bonded water molecules with ClO4^-^, the peak areas showed a significant difference with increasing perchlorate anion concentration and depending on the nature of the cations tested (Li, Na, and Mg). Moreover, Mozgawa et al. [37] using FTIR in the study of immobilization of HM (Pb^2+^, Cd^2+^, Ag^+^, and Cr^3+^) from aqueous solutions on various forms of natural sorbents, reported that the presence of HM cations in the structure of montmorillonite caused a modification in band intensity, due to the stretching vibrations of OH groups, revealing systematic changes related to the type of cation (its chemical nature) and its concentration in the initial solution. More recently, Kepenek et al. [20] reported that the areas under the curve of the peaks and bands, associated with the spectral regions being studied (proteins, carbohydrates), provided important information about the concentration of HM ions associated with the organic molecules studied. FTIR spectroscopy has been applied for a long time to study protein structures and functions, and also enzyme activity [46,56], including the interactions that HM exerts on proteins [57,58,59,60].

Figure 2a shows how the HM presence affects mainly the behavior of the outermost part at the OH band; for that Figure 3 shows this behavior for the OH bands at 3400 and 1640 cm^−1^. In both figures was possible to observe how the OH spectra in the mid-infrared region (black line) present now several peaks by the HM presence, the most by Pb (light green) and the less by Cr (blue); the region more affected was the band at 1640 cm^−1^. Similar behavior was observed by Ning Y et al. in 2012, for five HM at different concentrations, the difference was that authors sampled in the near-infrared region; and absorbance differences associated with metals were more notable, this was by they used hydroxyapatite as adsorbent and diffuse reflectance mode for sampling [61], so the libration mode never appear in their spectrum. In 2016 Rauh F and Mizaikoff B showed how the monoatomic ions affect the spectral behavior of OH bands in the mid-infrared region in the function of temperature; to generate calibration functions, the spectra data for the bands to 3350 and 1640 cm^−1^ were mathematical processing to generate linear regressions [62].

FTIR spectroscopy was reported as far back as 1996 [58], and more recent works continue to make use of this tool to study OH interactions in several research contexts [20,30,38,51,54,57,58,59,60,63]. We obtained the spectra of β-casein solutions with Cd^2+^, Cr^6+^, and Pb^2+^ at different concentrations, showing that absorbance intensities in the hydroxyl and amide regions increase in proportion to HM concentrations, possibly caused by interactions between nucleophilic groups such as -COOH, -OH, and -NH_2_ and the presence of cations (Figure 4 and Figure 5). Casein is a very flexible protein [48,64,65,66]. It presents a high percentage of Pro, Gln, Leu, Val, and Glu and a meager portion of Gly, Arg, and Asp in its sequence, compared to the global amino acid composition in mammal proteins [67,68] (Table 1). Casein does not present a unique and defined three-dimensional structure, nor even clear hydrophobic or hydrophilic domains. At temperatures below 15 °C, casein remains in a monomeric state. The formation and size of the micelles are directly proportional to temperature, up to 30 °C above its Critical Micellar Concentration. Increasing temperature increases the flexibility of the casein, by exposing functional groups that remain hidden in the monomeric state [49,50]. Taken together, this has made it difficult for casein to crystallize in its native state. It has also led to the assumption that cationic ions, such as Cd^2+^, Pb^2+^, or Cr^6+^ interact with any of the multiple Glu residues in casein, neutralizing their net charges and stabilizing the structure of micelles. This stabilized structure enables absorbance at the wavenumber observed. The more anions there are, the more protein portions will acquire the structure that is required for absorbing at the experimentally observed wavenumber. The interaction effect between nucleophilic residues and divalent cations has been published by Nara et al. [59]; this work explains how ions in the solvated state can generate coordinated structures with nucleophilic groups such as hydroxyl, carbonyls, carboxyl, amines, and protein amides. These clusters can be found in abundance in the residual amino acids of virtually all proteins, including β-casein. In a more complex matrix such as milk, similar behavior was observed in the protein region (Figure 7 and Figure 8); Gerbino et al. [57] reported interactions between Lactobacillus kefir S-layer proteins (CIDCA 8348 and JCM 5818) and Cd^2+^, Zn^2+^, Pb^2+^, and Ni^2+^ ions, using FTIR spectroscopy, after an analysis of amide I and amide II IR regions was undertaken, the authors reported that the amide II band shows maximum shifts from 1535 to 1495 cm^−1^, accompanied by a decrease in intensity, indicating that the metal/protein interaction occurs mainly due to the carboxylate groups of the side chains of the Asp and Glut residues, with some contribution from the NH groups that pertain to the peptide structure. They also noted changes in protein secondary structures, resulting from the interaction with those metal ions, observing a general tendency to increase the number of β-sheet structures and reduce the number of α-helices. These changes appear to enable proteins to adjust their structure in the presence of metal ions with minimal energy expenditure. These reports concur with our findings both in silico and experimental observations. Based on similar results and the use of multivariate methods, Kepenek et al. [20] were able to distinguish the microbial populations of strains that were exposed to Cd and Pb from those that were not, even at concentrations as low as 30 mgmL^−1^. Moreover, Sawalha et al. [60] reported that active groups such as hydroxyl, amino, carbonyl, carboxyl, and phosphates, present on the surface of microbial cells, are not only capable of binding HM (including Cr^6+^) through mechanisms such as complexation or chelation but these processes can also be observed using FTIR spectroscopy., Likewise even the materials tested in their study were able to promote oxidation of Cr^6+^ to Cr^3+^, which could also be observed using FTIR spectroscopy. This wholly concurs with the results presented in Figure 9, the HM binding varies in function of the sample composition, all ions present in the sample matrix may be affected by the HM presence, and absorption intensity was in function of the HM concentration.

In 2020 V. Kumar et.al. reported on how the presence of four HM affects Indian water bodies [69], but the most significant aspect of this work is the use of PCA to determine the function of the total PCA variance, that Pb and Zn have maximum loadings for PC1 and maximum loadings of PC2 on Cu and Cr. In our work, PCA revealed a clear clustering and tendency, in accordance with the matrix and HM concentration, showing a linear relationship between PC1 and PC2. This can be used in future works to predict HM concentrations, in the function of the interaction generated with the sample, as long as the following considerations are applied to our results: (a) the studies were carried out using pure compounds; (b) possible interferences between other ions such as Na^+^, Ca^2+^, Mg^2+^ among others commonly found in complex matrices such as milk, and the HM reported here, were not studied in this work.

## 5. Conclusions

HM ions such as Cd^2+^, Cr^6+^, and Pb^2+^ generate molecular interactions with anionic groups such as carboxyl, amides, and OH in water, casein, and milk. In the case of casein and milk, these interactions contribute to stabilizing the structure of the protein, meaning that the greater the presence (concentration) of ions, the greater the absorbance. Such interactions were visible in FTIR-ATR spectra, in regions similar to those reported by various authors for cations, including the HM studied here.

The in silico analysis revealed that the interactions between HM and anionic groups such as COOH, OH, and NH_2_ present mainly in Gln, Hys, Glu, Pro, and Phe confer stability to the casein structure.

The chemometric analysis made it possible to discriminate between the nature of the metal and the type of matrix. Likewise, a quantitative relationship between PC1 and PC2 as a function of concentration with a value of r^2^ ≥ 0.95 was established, possibly indicating a useful model to quantify these metals in an aqueous and dairy matrix, using FTIR-ATR and PCA.

These findings show that it was possible to observe experimentally the interactions of the studied HM with casein present in milk and justified by in silico analysis. In addition, using PCA was possible to discriminate between the nature of metal and observe a trend between their concentrations. The above can be useful for the later development of a faster and non-destructive method to identify and quantify HM in milk and water based on FTIR that supports the monitoring of these metals in real-time in the food industry.

## Figures and Tables

**Table 1:** Description of amide geometries allowing absorbance at experimentally observed wavenumber.

Wavenumber (cm^−1^)	Group	Description
1473	Gln, Hys radicals, and peptide bonds	When Gln or Hys radicals lie in the same plane as another amide, including peptide bonds that are antiparallel to each other, at a distance of 4.5 Å
1489	Gln or Glu radical with a peptide bond	When the radical amide of Gln and the peptide bond are at an angle ≈ 90° and at 4.5 Å or when the plane of the carboxyl radical of Glu and the peptide bond lie in the same plane
1508	Imide group of proline with any amide	Imide and the peptide bond on the same plane and another amide in parallel at 4.5 Å
1522	Glu, Gln radicals, and peptide bond	The amide planes are perpendicular to the plane of the carboxylic acid of Glu or to the plane formed by a C carbonyl, its O, and a sp3 C
1540	Peptide bond and radical amide from Gln	Two amides at an angle of 100° with the vertex being a C sp^3^ or the plane formed by a carbonyl, its O and a C sp^3^
1558	Peptide bond	When pairs of two antiparallel peptide bonds separated by a Cα from Val or Glu (such as a hairpin motif) are close to each other
1575	Peptide bond from Pro, radical benzene from Phe, and carboxylic from Glu	The benzene plane of Phe lies parallel to its C-Ter peptide bond and perpendicular to the next peptide bond, which is from Pro

**Table 2:** Selected regions for PCA in water spectra and justification for vibrating mode.

Region	Intervals in Wavenumber (cm^−1^)	Justification ^1^
1	3500–3000	Stretching of -OH
	2500–2000	Scissoring band
	2000–1500	Scissoring band
2	3500–3000	Stretching of -OH
	2000–1500	Scissoring band
3	3400–3200	Stretching of -OH
	2400–2250	Scissoring band
	1800–1450	Scissoring band
4	3400–3200	Stretching of -OH
	1800–1450	Scissoring band

^1^ Vibration modes in water [32,47].

**Table 3:** Selected regions for PCA in casein and milk spectra and justification for vibration mode.

Region	Intervals in Wavenumber (cm^−1^)	Justification ^2^
1	3500–3000	Stretching of -NH
	1600–1750	Carbonyl stretching
	2000–1500	Bending vibration -NH
2	3500–3000	Stretching of -NH
	1500–1300	C-N vibrations
3	3400–3200	Stretching of -NH
	2650–2000	-N-C-N- stretching
	1600–1250	Carboxylic acids, aromatics
4	3400–3200	Stretching of -NH
	1800–1450	Bending vibration -NH

^2^ Vibration modes by the functional group in casein [48,49,50].

## Data Availability

Data is contained within the article.
